# Heavy Metal Pollution and Ecological Assessment around the Jinsha Coal-Fired Power Plant (China)

**DOI:** 10.3390/ijerph14121589

**Published:** 2017-12-18

**Authors:** Xianfei Huang, Jiwei Hu, Fanxin Qin, Wenxuan Quan, Rensheng Cao, Mingyi Fan, Xianliang Wu

**Affiliations:** Guizhou Provincial Key Laboratory for Environment, Guizhou Normal University, Guiyang 550001, China; hxfswjs@gznu.edu.cn (X.H.); qinfanxin@126.com (F.Q.); wenxuanq@gznu.edu.cn (W.Q.); 18230825324@163.com (R.C.); fanmingyifmy@163.com (M.F.); wuxianliang1995@163.com (X.W.)

**Keywords:** heavy metals, coal-fired power plant, bio-accumulation, source assessment

## Abstract

Heavy metal pollution is a serious problem worldwide. In this study, 41 soil samples and 32 cabbage samples were collected from the area surrounding the Jinsha coal-fired power plant (JCFP Plant) in Guizhou Province, southwest China. Pb, Cd, Hg, As, Cu and Cr concentrations in soil samples and cabbage samples were analysed to study the pollution sources and risks of heavy metals around the power plant. The results indicate that the JCFP Plant contributes to the Pb, Cd, As, Hg, Cu, and Cr pollution in nearby soils, particularly Hg pollution. Cu and Cr in soils from both croplands and forestlands in the study area derive mainly from crustal materials or natural processes. Pb, Cd and As in soils from croplands arise partly through anthropogenic activities, but these elements in soils from forestlands originate mainly from crustal materials or natural processes. Hg pollution in soils from both croplands and forestlands is caused mainly by fly ash from the JCFP Plant. The cabbages grown in the study area were severely contaminated with heavy metals, and more than 90% of the cabbages had Pb concentrations exceeding the permissible level established by the Ministry of Health and the Standardization Administration of the People’s Republic of China. Additionally, 30% of the cabbages had As concentrations exceeding the permissible level. Because forests can protect soils from heavy metal pollution caused by atmospheric deposition, close attention should be given to the Hg pollution in soils and to the concentrations of Pb, As, Hg and Cr in vegetables from the study area.

## 1. Introduction

Heavy metals are widely used in the industrial and residential sectors due to their useful properties, such as their strength, malleability, and heat and electrical conductivity [1]. The demand for metals has increased with social development. Consequently, the metal uptake by crops and vegetables grown for human consumption has increased [2,3]. Heavy metals have high densities and are toxic or poisonous at low concentrations. The excess consumption of non-essential trace elements such as arsenic (As) and cadmium (Cd), even at relatively low levels, can cause various diseases, renal dysfunction, endocrine disruption, reproductive dysfunction, and cancers [4,5,6]. Heavy metals may enter the soil through bedrock or from anthropogenic by-products such as solid or liquid waste deposits, agricultural inputs, and industrial and urban emissions [7]. Soil contaminated with metals is a primary source of toxic element exposure to humans. Toxic metals in soils can enter the human body through the consumption of contaminated food crops or water or the inhalation of dust [8,9]. The presence of heavy metals in the soil is an important indicator of environmental pollution [10] and has become a serious issue worldwide.

Increased fossil fuel combustion during the past century is responsible for the progressive change in atmospheric composition [11,12,13]. Coal-fired power plants represent one of the most important anthropogenic sources of heavy metal pollution due to their tremendous annual coal consumption [14,15]. The release of metals from coal-fired power plants and their subsequent deposition in soil are well known to significantly alter the environmental quality of surrounding areas [16]. In 2012, the global coal consumption of coal-fired power plants was approximately 1785.3 million tons [17]. Heavy metals in coal can be distributed in solid and gaseous products, accumulating in the form of coal ash [18]. Some of the ash is released into the atmosphere through stacks and transferred into soils and waters by wet or dry deposition. To fulfil the demands of society and industrial development, coal-fired power plants will continue to play an important role in electric power generation. However, the amount of fly ash released by coal plants in the United States reached 72 million tons in 2006 [19]. Numerous studies of heavy metal pollution caused by coal-fired power plants have been conducted during the past several decades. Xu et al. conducted a study of the impact of a coal-fired power plant on the inorganic mercury and methyl-mercury distributions in rice, finding that the concentrations of MeHg and Hg(II) in rice samples collected adjacent to a coal-fired power plant were as high as 3.8 μg·kg^−1^ and 22 μg·kg^−1^, respectively. The Hg (THg) concentration of rice samples collected adjacent to a coal-fired power plant (24 μg·kg^−1^) exceeded the Chinese national standard limitation of 20 μg·kg^−1^ for THg in cereals [20]. Smołka-Danielowska found that the average concentrations of Cu, lead (Pb), chromium (Cr) and Cd in fly ash created during coal combustion at the Rybnik Power Station in Upper Silesia, southern Poland, were as high as 38 mg·kg^−1^, 44 mg·kg^−1^, 64 mg·kg^−1^, and 3 mg·kg^−1^, respectively [21]. In flat areas, migration of pollutants caused by coal-fired power plants depends on mainly the climate, particularly the wind direction, whereas the process is more complex in mountainous areas.

Guizhou Province is rich in coal. Electricity from coal-fired power plants in Guizhou Province satisfies the requirements of local cities and industries and is an important supplement in some eastern developed cities, such as Guangdong, Shanghai, Jiangsu, and Nanjing. Coal-fired power plants have been a powerful driver of the economic development of Guizhou Province for the past two decades, during which time the environment became severely polluted. The main objectives of this study are as follows: (a) to evaluate the contamination of soils by heavy metals in the area surrounding the Jinsha Coal-Fired Power Plant (JCFP Plant); (b) to explore the source of heavy metal in soils; and (c) to determine the heavy metal contamination of cabbages in the surrounding area of the JCFP Plant.

## 2. Materials and Methods

### 2.1. Study Area and Sampling

#### 2.1.1. Study Area

**Figure 1:**
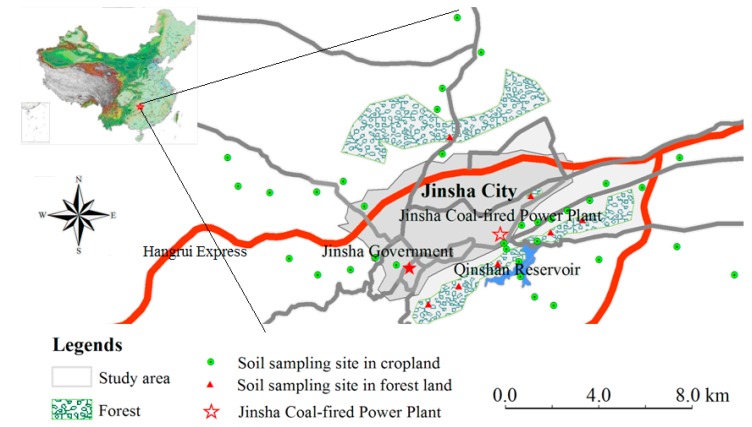
Distribution of sampling sites in the study region.

The JCFP Plant (27°28’29” N, 106°15’34” E) is located on the edge of Jinsha City in Jinsha County, Guizhou Province, southwestern China (Figure 1). The JCFP Plant includes eight units with a total installed capacity of 1700 MW. The plant uses coal from coal mines in the adjacent area, such as Jinsha, Qianxi, and Zunyi. The study region is a typical mountainous area, with mountains and hills comprising 92.50% of the total area. The climate of Jinsha County is subtropical humid monsoon. The mean annual temperature (MAT) and mean annual precipitation (MAP) range between 15 and 16 °C and between 800 and 1000 mm, respectively. The main ecosystem types are evergreen broad-leaved forest, coniferous and broad-leaved mixed forest, and montane elfin forest. The main tree species around the JCFP Plant are *Pinus massoniana* Lamb, *Platycladus orientalis* L. Franco, *Cryptomeria fortunei Hooibrenk ex* Otto et Dietr, *Camptotheca acuminata* Decne, and *Cyclobalanopsis glauca* (Thunb.) Oerst. Additionally, the main shrub species are *Rubus corchorifolius* L. F., *Viburnum dilatatum* Thunb, *Zanthoxylum simulans* Hance, *Trachycarpus fortunei* (Hook.) H. Wendl., *Millettia wight* et Arn., and *Pyracantha fortuneana* (Maxim.) Li. The main herbs are *Tuber sword* Fern, *Imperata cylindrica* Linn. Beauv, *Lobelia seguinii* Levl. Et Vant., *Herba artimisiae* Sieversianae, and *Herba Acroptili* Repentis.

#### 2.1.2. Field Sampling

In 2015, soil samples were collected from 41 locations (34 sampling sites in croplands and 7 sampling sites in forestlands) around the JCFP Plant (Figure 1). Vegetable samples (cabbage) were collected from 32 locations where soil samples were also drawn (no cabbage was found at two of the 34 sampling sites in croplands). Soil samples were collected to a depth of 20 cm, and the locations were recorded using a handheld global positioning system (GPS). Soil samples were collected with a stainless steel shovel and immediately packed in self-zip plastic bags. To avoid cross contamination, the shovel was brushed and then flushed with soil from the subsequent sampling site. Each soil sample included mixed soil from four or five plots at each location. Simultaneously, three or four cabbages were sampled and mixed to form the vegetable sample.

### 2.2. Analytical Methods

The soil samples were air dried at room temperature in the laboratory and then homogenized and passed through a 2 mm sieve (preparation for determining the soil properties) after drying to a constant weight. Finally, the soil samples were ground in an agate mortar and passed through a 0.14 mm sieve for heavy metal analysis. The cabbage samples were dried in an air-blowing thermostatic oven after washing with deionized water prepared using a water purification system (Nex Power 2000 from Human Corporation, Seoul, Korea). Simultaneously, approximately 10 g of each plant sample was weighed and dried to a constant weight at 45 °C for water content analysis. Soil samples used for determining heavy metal content were digested according to United States Environmental Protection Agency (USEPA) procedures [22]. Pb, Cd, Cu and Cr concentrations were determined using inductively coupled plasma atomic emission spectroscopy (5300 V, Perkin Elmer Corporation, Waltham, MA, USA). Hg and As concentrations were determined using atomic fluorescence spectrometry (AFS-933, Jitian Corporation, Shanghai, China). The plant samples for metal content determination were digested with 4 mL of concentrated nitric acid (HNO_3_) and 1 mL of hydrogen peroxide (H_2_O_2_) using a microwave digestion system. The total content of the studied elements in plant samples was determined using an atomic absorption spectrometer (ZEEnit 700P, Jena Corporation, Jena, Germany). Analytical blanks were processed for all determinations. The analytical procedures used to determine heavy metals in soil samples and cabbage samples were assessed for quality control using certified reference materials GBW-07403 and GBW10020. The uncertainty of the analytical procedure was within 10%.

### 2.3. Quantification of the Soil Pollution Level

To assess the contamination level and determine the anthropogenic effect on heavy metals in the soils of the study region, the enrichment factor (*EF*), contamination factor (*CF*) and geo-accumulation index (*I_geo_*) values were calculated.

#### 2.3.1. Enrichment Factors

The extent of heavy metal contamination in the soils of the study area was assessed based on the determined concentrations and the baseline values of heavy metal concentrations in Guizhou Province. The enrichment factor of each element was obtained with Equation (1), which was modified from Ćujić et al. [18]:

(1) $$EF_{M}=\frac{\left([M]/{\left[Fe\right]}_{Stu}\right)}{\left([M]/{\left[Fe\right]}_{Gui}\right)}$$

where *EF_M_* is the enrichment factor of element *M*, [*M*] is the concentration of the element *M*, and [*Fe*] is the concentration of iron. The subscripts “*_Stu_*” and “*_Gui_*” indicate the concentrations of Fe in the studied area and Guizhou Province, respectively. Here, Fe was used as the reference element for geochemical normalization because it is associated with fine solid surfaces, its geochemistry resembles that of many heavy metals, and its natural concentrations tend to be uniform [18,23]. According to previous studies, five contamination categories can be created based on the enrichment factor, as specified in Table 1 [24,25].

#### 2.3.2. Contamination Factors

The *CFs* were obtained by dividing the determined value by the baseline value (Equation (2)):

(2) $$CF=\frac{C_{Stu}}{C_{Gui}}$$

where *CF* is the contamination factor, *C_Stu_* is the heavy metal concentration in the soil sample, and *C_Gui_* is the baseline in Guizhou Province. Based on their intensities, the contamination levels were classified on a scale from 1 to 6, with the highest classification (6) indicating that the metal concentration is 100 times greater than the level expected in the Earth’s crust [18].

#### 2.3.3. Geo-Accumulation Index

*I_geo_* was used to evaluate the degree of heavy metal pollution in the soils from the study area. This index was calculated with Equation (3):

(3) $$I_{geo}=\log_{2}(\frac{C_{Stu}}{1.5\times C_{Gui}})$$

where *I_geo_* is the geo-accumulation index, 1.5 is the background matrix correction factor introduced to account for possible differences in the background values due to lithospheric effects, *C_stu_* is the heavy metal concentration in the soil sample, and *C_Gui_* is the baseline concentration in Guizhou Province. Seven classes were established based on *I_geo_*: *I_geo_* < 0, class 1 (uncontaminated to moderately contaminated); 0 < *I_geo_* < 1, class 2 (moderately contaminated): 1 < *I_geo_* < 2, class 3 (moderately to heavily contaminated); 2 < *I_geo_* < 3, class 4 (heavily contaminated); 3 < *I_geo_* < 4, class 5 (heavily to extremely contaminated); 4 < *I_geo_* < 5, class 6 (extremely contaminated); 5 < *I_geo_*_,_ with class 7 being an open class that comprises all values of the index higher than class 6. The heavy metal concentrations in class 7 may be 100 times greater than the geochemical background value [18,26].

#### 2.3.4. Quantification of the Potential Risk to Humans

To evaluate the potential risks of heavy metals to humans in the study region, the accumulation factors (*AFs*) of the studied heavy metals in cabbage were determined. *AFs* were calculated using Equation (4):

(4) $$AF=\frac{C_{cabbage}}{C_{soil}}$$

where *C_cabbage_* is the heavy metal content in cabbage (dry weight), and *C_Soil_* is the heavy metal content in the soil. Here, *AF* < 1 suggests that cabbage the specific element does not accumulate in cabbage; 1 < *AF* < 2 reflects low accumulation; and *AF* > 2 reflects high accumulation [27].

### 2.4. Statistical Methods

Data processing and statistical analysis were performed using Microsoft Excel 2003 (Microsoft, Redmond, WA, USA), the Statistical Package for the Social Sciences version 18.0 (SPSS 18.0, IBM, Armonk, NY, USA), and ArcGIS mapping software (ArcMap 10.3, ESRI, Redlands, CA, USA).

## 3. Results and Discussion

### 3.1. Heavy Metal Pollution in the Soil

#### 3.1.1. Heavy Metal Content and Properties of the Soils from the Study Area

The descriptive statistics of the analysed heavy metals and the soil properties of soil samples from 41 locations in croplands around the JCFP Plant are summarized in Table 2.

The mean concentrations of Pb, Cd, Hg, Cu, As and Cr in soil samples from croplands around the JCFP Plant were 46.02, 0.62, 0.70, 26.40, 35.51 and 52.62 mg·kg^−1^, respectively. Additionally, the mean concentrations of Pb, Cd, Hg, Cu, As and Cr in soil samples from forest areas around the JCFP Plant were 33.66, 0.58, 0.30, 11.99, 27.98 and 31.66·mg kg^−1^, respectively. Moreover, the mean pH in soil samples from croplands was slightly lower than that in soil samples from forest areas. Notably, all of the mean concentrations of heavy metals in soil samples from croplands around the JCFP Plant are higher than those in soil samples from forests. In addition, the mean values of nutrient elements, such as total nitrogen (TN), total phosphorus (TP), organic matter (OM), and total organic carbon (TOC), in soil samples from croplands were much lower than those in soil samples from forests.According to the National Soil Resource Survey of China, the baseline levels of Pb, Cd, Hg, As, Cu and Cr in Guizhou Province are 35.20, 0.66, 0.11, 20.00, 32.00 and 95.50 mg·kg^−1^, respectively [28]. Generally, soils from croplands in the study area were polluted by Pb, Hg, As and Cu. The mean levels of the studied heavy metals, except for Hg, in the soils from forestlands were lower than the baseline levels of heavy metals in Guizhou Province. However, this conclusion is based only on mean values. For example, the Pb content in soils from 10 sampling sites located in croplands was lower than 35.20 mg·kg^−1^, and the minimum value at these sites was 4.73 mg·kg^−1^. Conversely, the Pb content in soils from two sampling sites located in forest areas was higher than 35.20 mg·kg^−1^, and the maximum concentration reached 39.01 mg·kg^−1^.

#### 3.1.2. Spatial Distributions of Heavy Metals in Soils from the Study Area

**Figure 2:**
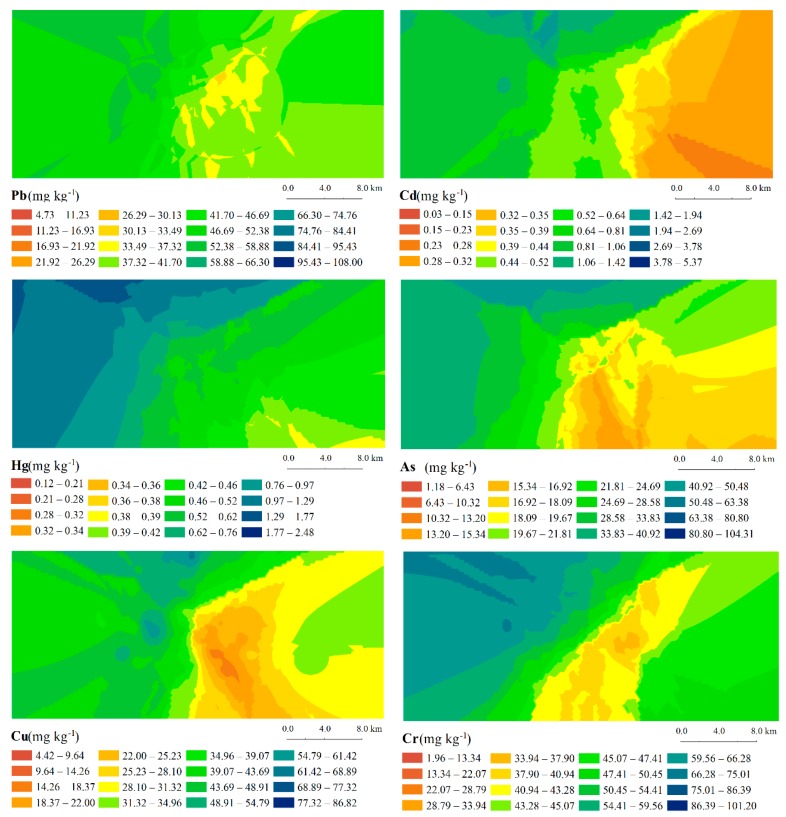
Spatial distributions of heavy metal concentrations in soils from the study area obtained with empirical Bayesian kriging interpolation.

The spatial distributions of the studied heavy metals in the soils from the study area were obtained by empirical Bayesian kriging interpolation and are shown in Figure 2.

The spatial characteristic maps illustrate high variations in the heavy metal content of the soils, except for Pb. However, the other heavy metals exhibit certain similar spatial characteristics. The heavy metal concentrations in soils collected from the southeastern, eastern and northeastern parts of the studied area are generally lower than those in soils collected from the southwestern, western and northwestern parts. Additionally, no heavy industry exists in the study region. The wind in the study area blows mainly from the northeast and southeast directions. The northeast wind is often re-directed by the mountains in the southern part of the study area, becoming a southeast wind. Consequently, large amounts of fly ash created by the JCFP Plant are deposited in the northwestern region of the study area. We believe that the JCFP Plant contributes little to Pb pollution in soils in the study area. The Pb pollution in soils from the study area is mainly associated with agricultural activities, such as fertilization and pesticide application. However, the JCFP Plant is an important source of other heavy metals (Cd, Hg, As, Cu and Cr) in the soil.

#### 3.1.3. Principal Component Analysis of Heavy Metals in Soils from the Study Area

**Figure 3:**
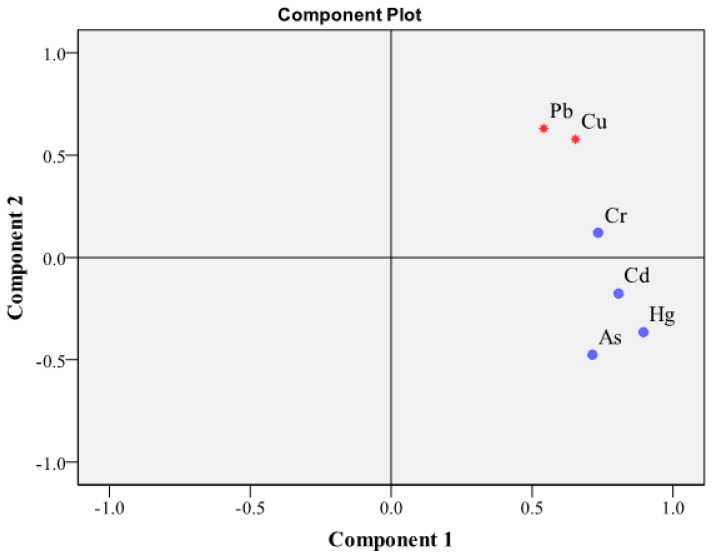
Principal component analysis of the heavy metal concentrations in soils from the study area.

A principle component analysis (PCA), a type of multivariate statistical analysis, was performed based on the heavy metal distributions. Two components were extracted, and the results are shown in Figure 3. All the studied heavy metals exhibited significant positive correlations with component 1. The loading scores of Pb, Cd, Hg, As, Cu and Cr for component 1 were 0.54, 0.81, 0.90, 0.71, 0.65 and 0.73, respectively. Additionally, Pb and Cu exhibited significant positive correlations with component 2, with loading scores of 0.63 and 0.68, respectively. As noted, no heavy industry exists in the study area, and the JCFP Plant is the only significant source of point pollution. Therefore, component 1 likely represents pollution from the JCFP Plant, and component 2 represents the pollution caused by agricultural activities. The PCA analysis results suggest that the distributions of Pb and Cu in soils from the study area are likely associated with the combined actions of the JCFP Plant and agricultural activities, and the Cd, Hg, As and Cr levels and distributions are mainly associated with the activities of the JCFP Plant.

#### 3.1.4. Indices of Heavy Metal Pollution in Soils from the Study Area

The *EF* ranges of Pb, Cd, Hg, As, Cu and Cr in soils from croplands in the study area were 0.02–1.98, 0.02–8.14, 0.77–16.97, 0.08–2.42, 0.05–1.43 and 0.11–1.06, respectively (Table 3). The *EF* ranges of Pb, Cd, Hg, As, Cu and Cr in soils from forest areas were 0.30–0.76, 0.18–0.84, 0.36–1.98, 0.02–0.89, 0.08–0.82, and 0.01–0.23, respectively. Based on the mean *EFs*, the enrichment of heavy metals in soils from croplands in the study area can be classified as no enrichment to minimal enrichment, except for Hg, which exhibited moderate enrichment. The enrichment of heavy metals in soils from forest areas can be classified as no enrichment to minimal enrichment. Notably, all the mean *EF* values of heavy metals in soils from croplands are higher than those of heavy metals in soils from forestlands. This result suggests that forests are likely effective shields, protecting soils from pollution caused by atmospheric deposition. Previous studies have suggested that metals are derived from mainly crustal materials or natural processes if their *EF* values are between 0.05 and 1.50 and likely from anthropogenic activities if the *EF* values are higher than 1.5 [18,29]. All the *EFs* of Cu and Cr in soils from croplands in the study area were within the range of 0.05 and 1.5. Thus, Cu and Cr derived from mainly crustal materials or natural processes in the study area. The *EFs* of Pb, Cd, Hg and As in soils collected from some the sampling sites in croplands were higher than 1.50. Therefore, anthropogenic activities are non-ignorable sources of heavy metal pollution in the study area with respect to these elements, particularly Hg, as 73.52% of the *EFs* of Hg exceeded 1.50. The *EFs* of Pb, Cd, As, Cu and Cr in soils from forestlands were within the range of 0.05 to 1.5, but 57.14% of the *EFs* of Hg in soils from forestlands were greater than 1.50. In summary, the concentrations of the studied heavy metals in soils from croplands were generally higher than those from forestlands. The *EFs* values indicated that heavy metal pollution in soils from croplands was more extensive than that in soils from forestlands. Pb, Cd, As, Cu and Cr in soils from forestlands derived from mainly crustal materials or natural processes, whereas the Hg in soils from forestlands likely originated from atmospheric deposition.

The range of *I_geo_* values for Pb, Cd, Hg, As, Cu and Cr in soils from croplands in the study area were −4.76 to 1.03, −5.04 to 2.44, 0.40 to 3.91, −2.89 to 1.80, −3.17 to 0.85 and −2.09 to −0.50, respectively. The range of *I_geo_* values for Pb, Cd, Hg, As, Cu and Cr in soils from forestlands were −1.12 to −0.39, −1.46 to 0.06, −0.24 to 1.66, −4.67 to −0.06, and −6.19 to −0.89, respectively. Based on the mean values of *I_geo_* for Pb, Cd, As, Cu and Cr, the soils from both cropland and forestland areas could be categorized as class 1 (Table 3), meaning these soils are uncontaminated or moderately contaminated. However, the mean *I_geo_* value of Hg in soils from cropland was 1.81; thus, Hg exhibited moderate to heavy contamination in these areas (class 3). The mean *I_geo_* value of Hg in soils from forestlands was 0.79, suggesting that Hg pollution in soils from forestlands was moderate. Notably, all of the *I_geo_* values of Hg in soils from cropland were greater than 0, and five *I_geo_* values for Hg in soils from forestlands were greater than 0. Thus, Hg pollution is likely a significant problem in the study area. Some *I_geo_* values for Pb, Cd, As and Cu in soils from croplands were greater than 0. Specifically, all of the *I_geo_* values for Cr in soils from both croplands and forestlands were lower than 0, and all of the *I_geo_* values for Pb and Cu in soils from forestlands were lower than 0. In conclusion, Hg is the top-priority element among the studied elements in soils around the JCFP Plant.

The ranges of *CF* values of heavy metals in soils from croplands were as follows: Pb (0.06–3.07), Cd (0.05–8.14), Hg (1.98–22.59), As (0.20–5.22), Cu (0.17–2.71) and Cr (0.35–1.07). The ranges of *CF* values in soils from forestlands were as follows: Pb (0.69–1.14), Cd (0.55–1.56), Hg (1.27–4.73), As (0.06–1.56), Cu (0.14–1.60) and Cr (0.81–0.36). The mean *CF* values indicate that the soils from croplands around the JCFP Plant were contaminated with Pb, Hg, As and Cu, whereas the soils from forestlands were contaminated by mainly Cd and Hg.

In the present study, the *CF* values indicate that the soils are contaminated with certain metals, whereas the *EF* and *I_geo_* data suggest either no or moderate contamination of most metals. As shown in Equations (1)–(3), the concentrations of Fe in the studied area and Guizhou Province were employed to eliminate the effect of regional geochemical process, and the background matrix correction factor (1.5) was introduced to account for possible differences in the background values due to lithospheric effects in calculating the *I_geo_* data. However, the *CF* values were calculated by dividing the determined value by the baseline value. We believe that these values have their own advantages. The results from the *EF* and *I_geo_* data could be used for global comparisons. However, the results from *CF* values are more likely of local interest.

### 3.2. Quantification of the Potential Risks of Heavy Metals to Humans

#### 3.2.1. Heavy Metal Content of Cabbages

The mean values of Pb, Cd, Hg, As, Cu and Cr in cabbages (fresh weight) from the study area were 0.38, 0.07, 0.01, 0.06, 0.51 and 0.43 mg·kg^−1^, respectively. The descriptive statistics of heavy metal concentrations (mg·kg^−1^) in cabbages from the study area are shown in Table 4. To assess the risk of heavy metals in vegetables from the study area, the heavy metal content of the cabbage samples was compared with the “maximum levels of contaminants in foods” (fresh samples), namely, Pb (0.10 mg·kg^−1^); Cd (0.20 mg·kg^−1^); Hg (0.01 mg·kg^−1^); As (0.05 mg·kg^−1^) and Cr (0.50 mg·kg^−1^). These permissible values were established by the Ministry of Health and the Standardization Administration of the People’s Republic of China [30]. Approximately 97.06% of cabbages from the study area had Pb concentrations exceeding the maximum level; 8.82% of cabbages Cd concentrations exceeding the maximum level; 18.75% of cabbages had Hg concentrations exceeding the maximum level; 34.38% of cabbages had As concentrations exceeding the maximum level; and 18.75% of cabbages had Cr concentrations exceeding the maximum level. No information is available regarding the limit value of Cu. Therefore, heavy metal pollution of vegetables is a serious problem in the study area, and considerable attention should be given to these elements, particularly Pb.

#### 3.2.2. Accumulation Factor

Vegetables can absorb and accumulate heavy metals at concentrations sufficiently high to cause clinical problems in both animals and humans [31]. Moreover, the same vegetable can accumulate Pb, Cd, Hg, As, Cu and Cr differently. The results of this study indicate that cabbage accumulates different elements at different rates (Table 5). The *AF* of Pb ranged from 0.02 to 0.77, with a mean valve of 0.18; that of Cd ranged from 0.02 to 8.39, with a mean value of 1.85; that of Hg ranged from 0.01 to 0.54, with a mean value of 0.15; that of As ranged from 0.00 to 0.27, with a mean value of 0.03; that of Cu ranged from 0.05 to 1.21, with a mean value of 1.85; and that of Cr ranged from 0.02 to 0.56, with a mean value of 0.11. Based on these mean *AF* values, the accumulation ability of cabbage exhibited the following order for the studied elements: Cd > Cu > Pb > Hg > Cr > As. Only Cd slowly accumulated in cabbage.

## 4. Conclusions

The sources and levels of heavy metal pollution in the study area were investigated by conducting a statistical analysis of heavy metals in 41 soil samples (34 from croplands and 7 from forestlands) and 32 cabbage samples collected around the JCFP Plant.

The results of the *EF* analysis indicate that Cu and Cr in soils from both croplands and forestlands in the study area derived from mainly crustal materials or natural processes. Pb, Cd and As in soils from croplands originated partly from anthropogenic activities, but these elements in soils from forestlands derived from mainly crustal materials or natural processes. Hg in soils from both croplands and forestlands resulted from mainly anthropogenic activities (fly ash generated by the JCFP Plant). The geo-accumulation index analysis indicates that no Cr exists in soils from the study area. However, Hg concentrations in soils in both croplands and forestlands are a severe problem. Moreover, Pb, Cd, As and Cu were observed in soils collected at some sampling sites in cropland areas, and Cd and As were observed in some soil samples collected from forestland areas.

In summary, the JCFP Plant is a point pollution source of Cd, Hg, As, Cu and Cr in soils around the power plant. Hg concentrations in soils around the JCFP Plant is a particularly serious problem. Pb deposition in soils from the study area is caused mainly by agricultural activities. Forests are effective shields, protecting soils from pollution caused by atmospheric deposition. Additionally, Cd was observed to accumulate in cabbage. Based on the “maximum levels of contaminants in foods”, the heavy metal content of cabbage around the JCFP Plant poses serious risks. Efforts should focus on ensuring the safety of vegetables for consumption by local residents.

## Figures and Tables

**Table 1:** Contamination categories based on the enrichment factor.

Enrichment Factor	Contamination Category
*EF* < 2	no enrichment to minimal enrichment
*EF* = 2–5	moderate enrichment
*EF* = 5–20	significant enrichment
*EF* = 20–40	very high enrichment
*EF* > 40	extremely high enrichment

*EF*: the enrichment factor.

**Table 2:** Descriptive statistics of the heavy metal concentrations (mg·kg^−1^) and soil properties of soil samples around the JCFP Plant.

Parameter	Pb	Cd	Hg	As	Cu	Cr	pH	TN (g·kg^−1^)	TP (mg·kg^−1^)	OM (%)	TOC (g·kg^−1^)
**Croplands**											
Median	50.02	0.37	0.70	24.55	30.94	51.92	6.70	1.36	254.31	6.45	22.30
Mean	46.02	0.62	0.70	26.40	35.51	52.62	6.71	1.35	277.27	4.02	23.20
Std. deviation	24.24	1.06	0.49	18.09	23.61	14.44	0.12	0.50	151.16	4.66	26.92
Range	103.27	5.34	2.27	100.27	81.50	67.61	0.28	1.22	420.42	12.13	70.01
Minimum	4.73	0.03	0.22	4.04	5.32	33.59	6.57	0.76	49.33	1.32	7.62
Maximum	108.00	5.37	2.49	104.31	86.82	101.20	6.85	1.98	469.75	13.45	77.63
Skewness	0.40	3.85	2.05	2.65	0.98	1.31	0.05	0.44	−0.51	2.35	2.35
Kurtosis	−0.15	15.01	5.14	10.70	0.23	2.76	−2.23	−1.64	−0.42	5.60	5.60
**Forestlands**											
Median	36.57	0.67	0.38	4.26	22.56	29.23	7.18	2.22	451.32	8.59	48.96
Mean	33.66	0.58	0.30	11.99	27.98	31.66	7.21	2.17	440.77	8.79	50.76
Std. deviation	5.54	0.40	0.15	13.39	19.40	25.23	0.14	0.52	64.55	4.22	24.37
Range	14.64	1.03	0.38	30.05	46.92	75.32	0.39	1.30	144.24	12.67	73.15
Minimum	24.37	0.00	0.14	1.18	4.42	1.96	7.06	1.32	368.29	2.41	13.92
Maximum	39.01	1.03	0.52	31.23	51.34	77.28	7.45	2.62	512.53	15.08	87.07
Skewness	−0.95	−0.22	0.29	0.98	0.06	1.24	1.04	−1.15	−0.02	−0.02	−0.02
Kurtosis	0.33	−1.05	−1.75	−1.61	−2.24	2.62	0.10	−0.25	−2.86	0.92	0.92

TN: total nitrogen; TP: total phosphorus; OM: organic matter; TOC: total organic carbon.

**Table 3:** Mean values of the pollution indices of soil samples around the JCFP Plant.

Pollution Indices	*EF*		*I_geo_*		*CF*	
	Croplands	Forestlands	Croplands	Forestlands	Croplands	Forestlands
Pb	0.64	0.48	−0.56	−0.63	1.29	0.98
Cd	0.57	0.52	−1.50	−0.67	0.93	1.04
Hg	3.26	1.40	1.81	0.79	6.34	2.90
As	0.64	0.28	−0.47	−2.36	1.32	0.54
Cu	0.53	0.39	−0.79	−1.19	1.11	0.85
Cr	0.27	0.16	−1.49	−2.61	0.55	0.36

**Table 4:** Descriptive statistics of heavy metal concentrations (mg·kg^−1^) in cabbages from the study area.

Parameter	Pb	Cd	Hg	As	Cu	Cr
**Fresh Weight**						
Mean	0.38	0.07	0.01	0.06	0.51	0.43
Std. deviation	0.21	0.10	0.00	0.12	0.16	0.44
Range	0.86	0.56	0.01	0.68	0.95	2.48
Minimum	0.08	0.01	0.00	0.00	0.21	0.12
Maximum	0.94	0.56	0.01	0.68	1.16	2.60
Skewness	1.01	3.91	0.00	4.86	2.07	4.01
Kurtosis	1.46	17.70	–0.05	26.12	8.92	18.20
**Dry Weight**						
Mean	4.19	0.74	0.08	0.67	5.65	4.75
Std. deviation	2.29	1.13	0.04	1.30	1.73	4.94
Range	9.60	6.17	0.15	7.59	10.56	27.59
Minimum	0.87	0.07	0.00	0.00	2.36	1.31
Maximum	10.47	6.24	0.15	7.59	12.92	28.90
Skewness	1.02	3.91	0.01	4.86	2.09	4.00
Kurtosis	1.46	17.70	–0.05	26.12	8.92	18.20

**Table 5:** Descriptive statistics of the accumulation factors of cabbage for different heavy metals (dry weight).

Parameters	Pb	Cd	Hg	As	Cu	Cr
Mean	0.18	1.85	0.15	0.03	0.29	0.11
Std. deviation	0.21	1.98	0.13	0.05	0.28	0.11
Range	0.75	8.37	0.54	0.27	1.16	0.54
Minimum	0.02	0.02	0.00	0.00	0.05	0.02
Maximum	0.77	8.39	0.54	0.27	1.21	0.56
Skewness	1.89	1.78	0.92	4.05	2.03	3.03
Kurtosis	2.52	2.98	0.44	19.60	3.75	10.25
